# Wild grapevines as rootstock regulate the oxidative defense system of *in vitro* grafted scion varieties under drought stress

**DOI:** 10.1371/journal.pone.0274387

**Published:** 2022-09-13

**Authors:** Fahad Nazir, Touqeer Ahmad, Saad Imran Malik, Mukhtar Ahmed, Muhammad Ajmal Bashir

**Affiliations:** 1 Department of Horticulture, PMAS-Arid Agriculture University, Rawalpindi, Pakistan; 2 National Center of Industrial Biotechnology, PMAS-Arid Agriculture University, Rawalpindi, Pakistan; 3 Department of Plant Breeding and Genetics, PMAS-Arid Agriculture University, Rawalpindi, Pakistan; 4 Department of Agronomy, PMAS-Arid Agriculture University, Rawalpindi, Pakistan; 5 Department of Agriculture and Forest Sciences (DAFNE), University of Tuscia, Viterbo, Italy; Huazhong Agriculture University, CHINA

## Abstract

The narrow genetic base of modern cultivars is becoming a key bottleneck for crop improvement and the use of wild relatives is an appropriate approach to improve the genetic diversity of crops to manage the sustainable production under different abiotic and biotic constraints. In Pakistan, wild germplasm of grapevine viz Dakh, Toran, and Zarishk belong to *Vitis vinifera* subsp. *sylvestris* and Fatati belong to *Vitis vinifera* subsp. *sativa* is naturally present in humid and sub-humid areas of mountainous and sub-mountainous regions and showed varying level of tolerance against drought stress but have not been evaluated as rootstock. In this study, different tolerant behavior of wild grapevines as rootstock in grafted scion varieties were explored under different levels of PEG-6000 mediated drought stress i.e., -4.00, -6.00, and -8.00 bars. In response to drought stress, wild grapevines evoked several non-enzymatic and enzymatic activities. Among non-enzymatic activities, total chlorophyll contents of commercial varieties were sustained at higher level when grafted on wild grapevines Dakh and Fatati which subsequently reduced the damage of cell membrane via MDA. Whereas, to cope the membranous damage due to excessive cellular generation of ROS, wild grapevines triggered the enhanced activities of SOD to dismutase the free oxygen radicals into H_2_O_2_, then CAT enzyme convert the H_2_O_2_ into water molecules. Higher accumulation of ROS in commercial scion varieties were also coped by wild grapevines Dakh and Fatati through the upregulation of POD and APX enzymes activities. Based on these enzymatic and non-enzymatic indices, biplot and cluster analysis classified the wild grapevines as rootstock into three distinct categories comprises on relatively tolerant i.e., Dakh (*Vitis vinifera* subsp. *sylvestris*) and Fatati (*Vitis vinifera* subsp. *sativa*), moderate tolerant i.e., Toran (*Vitis vinifera* subsp. *sylvestris*) and relatively susceptible category i.e., Zarishk (*Vitis vinifera* subsp. *sylvestris*).

## Introduction

Grapevine is a commercial fruit crop of high economic importance and drought stress severely affected the production and quality of grapes [1, 2]. Important morpho physiological and biochemical responses of vines are altered by water stress through less water use efficiency, stomatal regulation, and higher generation of reactive oxygen species (ROS) due to the restricted transport of electron from cytoplasm to the mitochondria [3, 4]. Under these drought stress conditions, it is necessary to develop tolerant rootstocks that make grapevine cultivation sustainable by enhancing the oxidative activities under different environmental changes. Wild grapevine species and their hybrid genotypes are well known for being utilized as rootstock to make vine production sustainable under abiotic and biotic stresses due to the presence of inherited genetic tolerant traits [5]. Wild grapevine specie *V*. *vinifera* subsp. *sylvestris* is recognized as suitable for calcareous soils, *V*. *riparia* for winter hardiness, and *V*. *berlandieri* for chlorosis [6, 7]. Moreover, wild species of Asia like *V*. *jacquemontii*, *V*. *coignatiae*, *V*. *romanetii*, *V*. *yenshanensis*, and *V*. *betulifolia* have been found as resistance against powdery mildew and Botrytis cinerea and being originated from China, Iran, and Afghanistan [8, 9]. Among diverse wild grapevine germplasm, most of the drought tolerant rootstocks are derived by incorporating key resistant traits from wild *Vitis* species i.e., *berlandieri*, *riparia*, and *rupestris* [10]. Grapevine drought tolerant Ritcher 110 rootstock is originated from the cross of wild *rupestris* and *berlandieri* species which efficiently reduce the effect of drought stress on grafted scion varieties due to their well branched root system associated with the activation of differentially expressed genes such as expansin-like B1, xyloglucan endotransglucosylase and also auxin related proteins i.e., ROPs1 in the companion cell of the root phloem to assist the more water and nutrient uptake under severe drought stress condition [11]. These morphological attributes i.e., larger, and deeper root system of drought tolerant wild derived Ritcher 110 rootstock also make grafted vines productive under drought stress conditions by adopting different stress avoidance physiological mechanisms such as stomatal regulation, gaseous exchange, and net transpiration rate to adjust scion osmotically in changing irrigation regimes [12]. Moreover, physiological response such as stomatal conductance and less water loss from the leaf surfaces are also controlled by drought tolerant wild grapevine *V*. *riparia* rootstock through the endogenous production and over accumulation of ABA which can be upregulated through the expression of 9-cis-epoxycarotenoid dioxygenase (NCED) gene in the rootstock roots [13].

Apart from the morphological and physiological responses, wild hybrid rootstocks also modulate different enzymatic and non-enzymatic activities to elevate the toxic effect of osmotic stress usually caused by production of ROS. Excessive accumulation of ROS in scion leaf tissues are reduced by wild originated grapevine rootstocks Ritcher 110 and Ruggeri 140 with the help of biochemical defensive mechanisms such as catalase, peroxidase, superoxide dismutase and ascorbate peroxidase activities [14]. Moreover, enzymatic activities of catalase and glutathione reductase are also increased significantly by drought tolerant grapevine M4 rootstock [(*V*. *vinifera x V*. *berlandieri*) x *V*. *berlandieri* cv. Resseguier no. 1] through the over expression of “glutathione transferase GST 23-like isoform 1” and “glutathione S-transferase zeta class-like isoform 1” proteins to quench the excessive accumulation of dangerous free radicals [15]. Besides stress responsive proteins under drought stress condition, several lipase and lipoxygenase isoforms (LOXs) transcript can also be upregulated by wild originated drought tolerant grapevine rootstocks which helps in the metabolism of lipids through the synthesis of jasmonic acid (JA) that has been widely linked with the similar responses against abiotic stresses [16].

Selection of suitable grapevine varieties having adaptive features and superior quality traits is highly desirable for grape industry [17]. For instance, introduction of early maturing cultivars e.g., King’s Ruby and Flame Seedless changed the traditional cropping system in Pothawar [18]. These varieties usually ripen in June before the start of monsoon rains and give high fruit yield with more economic returns. Previously, these cultivars were grown as self-rooted crop in this region, and drought stress reduced the true potential of these cultivars. Exploitation of tolerant rootstocks from the available wide range of genetic variability is a sustainable approach to improve the drought resistance and to circumvent the negative effect of climatic changes. In Pakistan, wild grapevine germplasm locally known as Dakh, Toran, and Zarishk belongs to *Vitis vinifera* subsp. *sylvestris*, and Fatati into *Vitis vinifera* subsp. *sativa* is naturally present in humid and sub-humid areas of mountainous and sub-mountainous regions [19, 20]. According to several morpho-physiological and biochemical responses, these wild grapevines were screened for their different level of tolerance against in vitro PEG-6000 induced drought stress [21]. Until now, these native drought tolerant wild grapevines are not explored as rootstock, therefore, in present study, different tolerance level of wild grapevines as rootstock with commercial scion varieties were validated which will help in the sustainability of grapevine production in different grape growing regions prevailing drought stress conditions.

## Materials and methods

### Plant material, growing conditions, and drought stress induction

Wild grapevines commonly known as Dakh, Toran and Zarishk belongs to *Vitis vinifera* subsp. *sylvestris* and Fatati belongs to *Vitis vinifera* subsp. *sativa* were collected from dry and humid mountainous and sub-mountainous habitats of Pakistan. Nodal segments of wild grapevines and commercial scion varieties Kings Ruby and Flame Seedless were taken from *in vitro* conserved stock cultures of Plant Tissue Culture Laboratory and multiplied on shoot proliferation media [21].

For in vitro cleft grafting, “V” shaped upper portion (1.5 cm) of commercial varieties Kings Ruby and Flame Seedless containing one to two leaves were used as scion and inserted into vertical cut of basal portion (2 cm) of wild grapevines being used as rootstock. Sterilized aluminum foil was used to hold graft union at place and cultured on half strength MS medium (1/2 strength macro, micro elements, vitamins, sucrose, pH 5.8 and 6.75 g l^-1^ agar) for three weeks to promote graft compatibility between wild grapevines and commercial scion varieties. After three weeks of compatibility, grafted plants with trimmed roots and well developed 2–3 leaves were transferred on half strength MS medium supplemented with different levels of PEG-6000 infused drought stress i.e., -4, -6 and -8 bars. PEG solution (1:1) was prepared for osmotic potential determination and added by diffusion-based method [22] in different concentrations. All grafted and un-grafted plants subjected to different levels of drought stress were kept for three weeks under controlled conditions with temperature adjusted at 25 ± 1°C and light intensity at 2000 lux for 16/8 hours photoperiod with white fluorescent bulbs (Tuff ES 25W/56).

Experiment was replicate thrice and five plants per replication was taken. After three weeks of stress induction, fully developed leaves of grafted and un-grafted plants were taken for enzymatic and non-enzymatic biochemical analysis.

### Non-enzymatic activities of un-grafted and grafted plants

#### Total chlorophyll contents

Estimation of total chlorophyll contents were done according to method described by Arnon et al. [23] along with some modifications. Fresh leaf samples (100 mg) were collected and ground in 10 ml of 80% acetone. Absorbance was read at 645 and 663 nm using spectrophotometer (Model sp3000 plus, Optima Japan).

#### Malondialdehyde contents

For the quantification of malondialdehyde contents, method of Okhawa et al. [24] was used with modifications. Plant material of 0.2 g was extracted in 4 ml of 5% trichloro acetic acid (TCA). Thiobarbituric acid (TBA) (4 ml) was added to test tube containing 1 ml of extracted supernatant. Mixture was heated in water bath for 25 mins at 96°C and centrifuged at 10,000 rpm for 5 min at 4°C. Absorbance of supernatant was checked at 532 nm using spectrophotometer (Model sp3000 plus, Optima Japan).

#### Proline contents

Free proline contents of grafted and ungrafted treated plants were measured according to the method of Bates et al. [25] with modifications. Fresh leaf samples of 0.25 g weight were extracted in 5 ml of 3% (w/v) sulphosalicylic acid (0.01g/ 0.5 ml) and homogenate was obtained through filtration using filter paper (Whatman No. 1). Toluene (4 ml) was added and left at room temperature for 30 mins. The supernatant was collected, and absorbance was read at 520 nm using spectrophotometer.

#### Glycine betaine content

The amount of glycine betaine was measured by method designed by Grieve and Grattan [26]. Plant material (leaf sample of 0.5 g) was grinded and poured in conical flask with 20 ml of deionized water. Then sample was filtered and diluted (1:1) with 2N H_2_SO_4_. Cold potassium iodide-iodine solution (0.20 ml) was added, and the reactants were stirred gently with a vortex mixture. The crystals separated from acid media in supernatant was dissolved in 9 ml of 1,2-dichloroethane in test tube using vortex mixture. After 2 h, the absorbance was measured at 365 nm.

### Enzymatic activities of un-grafted and grafted plants

#### Superoxidase dismutase (SOD) activity

Superoxidase dismutase activity was determined according to the Method of Abassi et al. [27] was used to determine the activity of superoxidase dismutase. The assay mixture (300 μL of enzyme extract, 50 mM KPO_4_ buffer (pH 7.0), 200 μL of NBT + EDTA + methionine buffer and 50 μL riboflavin stock) was added into 3 ml cuvettes and placed under dark condition for 10 min. The absorbance was read at 560 nm for 45 sec and 60 sec by spectrophotometer (Model sp3000 plus, Optima Japan). One unit SOD activity was expressed as enzyme in Ug^-1^ protein.

#### Catalase (CAT) activity

Enzymatic activity of catalase was quantified according to the method of Abassi et al. [27]. The assay mixture (300 μL) of enzyme extract, 2.6 ml of 50 mM KPO_4_ buffer (pH 7.0), and 0.4 ml of 15 mM H_2_O_2_ was added into 3 ml cuvettes and placed under dark condition for 10 min. The absorbance was read at 240 nm for 45 and 60 sec by using spectrophotometer (Model sp3000 plus, Optima Japan).

#### Peroxidase (POD) activity

Peroxidase activity was determined according to the method described by Abassi et al. [27] with slight modification. The assay mixture consisted of 15 mM NaKPO_4_ buffer having pH 6.0 and 100 μl enzyme extract, 1mM H_2_O_2_ and 0.1 mM guaiacol (O-methoxyphenol). The absorbance of assay mixture was read at 470 nm with the help of spectrophotometer (Model sp3000 plus, Optima Japan). Peroxidase activity was calculated over a 3 min period and expressed as Ug^-1^ protein.

#### Ascorbate peroxidase (APX) activity

Activity of ascorbate peroxidase was calculated by following the method of Nakano and Asada [28]. Briefly, the total volume of 2 ml of reaction mixture was taken in 3 ml glass cuvettes. This reaction mixture was comprised of 25 mM (pH 7.0) sodium phosphate buffer, 0.1 mM EDTA, 0.25 mM ascorbate, 1.0 mM H_2_O_2_ and 100 μl enzymes extract. H_2_O_2_ dependent oxidation of ascorbate was followed and decrease in the absorbance was read at 290 nm at 60 sec intervals for 10 min by spectrophotometer (Model sp3000 plus, Optima Japan).

#### Experimental design and statistical analysis

This study was designed as factorial experiment in completely randomized design (CRD) with three replicates. Wild grapevines, commercial scion varieties and three levels of PEG-6000 induced drought stress i.e., -4, -6 and -8 bars were considered as major factors. Data was statistically analyzed by using Analysis of variances (ANOVA) techniques and differences among treatment means were compared by using least significance difference (LSD) test at 5% probability level using Statistics 8.1 software. To select more tolerant wild grapevines as rootstock, biplot was drawn based on the first two principal component using XLSTAT software. Cluster analysis was used to group the different wild grapevines as rootstock using similarity level measurement and Euclidean distance and drawn by Minitab v 21.1.0 software.

## Results

### Regulation of non-enzymatic activities by wild grapevines in grafted commercial scion varieties

In the present research, response of grafted wild grapevines as rootstock with commercial scion varieties were evaluated under control, and -4, -6 and -8 bars of PEG-6000 induced drought stress. According to the results, it was concluded that there was a significant difference, at p<0.05%, for all evaluated enzymatic and non-enzymatic biochemical traits, wild grapevines, commercial scion varieties and different levels of drought stress (Tables 1 and 2). Biochemical responses were identified as key markers in the present study to evaluate the susceptibility and tolerance levels of wild grapevines as rootstock. Among different stress levels, -8 bars proved to be most effective level against stress responsive traits whereas -6 and -4 bars had relatively less effect.

**Table 1:** Analysis of variance for enzymatic and non-enzymatic biochemical traits of commercial scion variety Kings Ruby grafted on different wild grapevines under drought stress condition.

		Mean Squares							
Sources of variation	df	TCC	MDA	Proline	GB	SOD	CAT	POD	APX
Replication	2	0.213	0.00114	0.00074	0.003	0.55	0.00002	0.01	4.076E-06
Wild grapevines	4	448.171	1.12711	2.50458	7.1328	272.67	0.06156	326.68	0.08388
Treatment	3	614.213	2.95862	7.15435	17.9746	2877.55	0.27491	2414.09	0.19104
Wild grapevines x treatment	12	12.944	0.03516	0.51417	1.0446	9.11	0.00756	33.47	0.00594
Error	38	0.157	0.00364	0.00088	0.005	0.31	0.00004	0.05	9.985E-06

**Table 2:** Analysis of variance for enzymatic and non-enzymatic biochemical traits of commercial scion variety Flame Seedless grafted on different wild grapevines under drought stress condition.

		Mean Squares							
Sources of variation	df	TCC	MDA	Proline	GB	SOD	CAT	POD	APX
Replication	2	0.006	0.00328	0.00005	0.0002	0.00244	0.00002	0.05	0.00002
Wild grapevines	4	573.928	1.81910	1.93748	7.2032	310.685	0.05548	274.01	0.05929
Treatment	3	675.513	3.26437	6.13754	15.9702	2712.19	0.23216	2211.88	0.17469
Wild grapevines x treatment	12	10.175	0.04541	0.38822	1.1204	4.43972	0.00643	37.60	0.00549
Error	38	0.171	0.01123	0.00033	0.0009	0.26908	0.00003	0.05	0.00001

### Total chlorophyll contents

**Fig 1:**
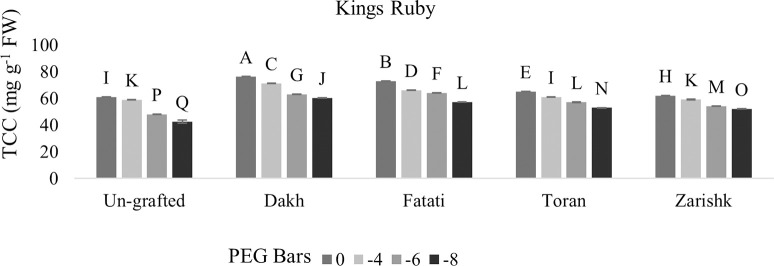
Total chlorophyll content (TCC) of un-grafted and grafted Kings Ruby scion varieties on different wild grapevines under drought stress condition. Data is represented as mean SD and letters showed significant difference at p<0.05.

**Fig 2:**
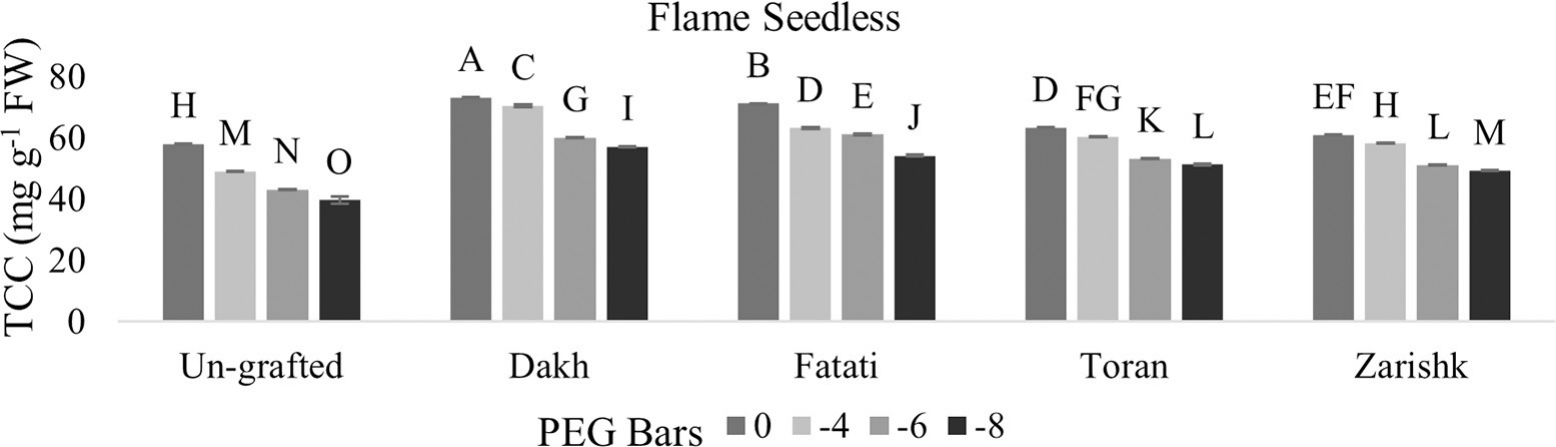
Total chlorophyll content (TCC) of un-grafted and grafted Flame Seedless scion varieties on different wild grapevines under drought stress condition. Data is represented as mean SD and letters showed significant difference at p<0.05.

Non-enzymatic activities such as total chlorophyll contents were controlled by tolerant rootstocks to survive the grafted scion varieties under drought stress conditions. When assessing wild grapevines as rootstock, significant interactions were observed between chlorophyll content of commercial varieties grafted on wild grapevines and progressive level of drought stress (Figs 1 and 2). In the context of maintained chlorophyll content, increased rate of susceptibility was observed in both the commercial scion varieties (Kings Ruby and Flame Seedless) at higher level of drought stress i.e., (-8 bars) due to the absence/defused structure of leaves. Therefore, it is inevitable to grow commercial grapevine varieties in drought affected areas with grafting at suitable rootstocks having capabilities to tolerate drought stress conditions. Commercial scion variety Kings Ruby exhibit 60 mg g^-1^ chlorophyll content whereas Flame Seedless showed 57 mg g^-1^ of chlorophyll content at higher level of drought stress when grafted on wild grapevine Dakh. At -6 bars of droughts stress decreased rate of chlorophyll content was observed in Flame seedless variety grafted on wild grapevine Zarishk, however, grafted Kings ruby showed relative increased chlorophyll content i.e., 54 mg g^-1^ when grafted on Zarishk.

### Malondialdehyde (MDA) contents

**Fig 3:**
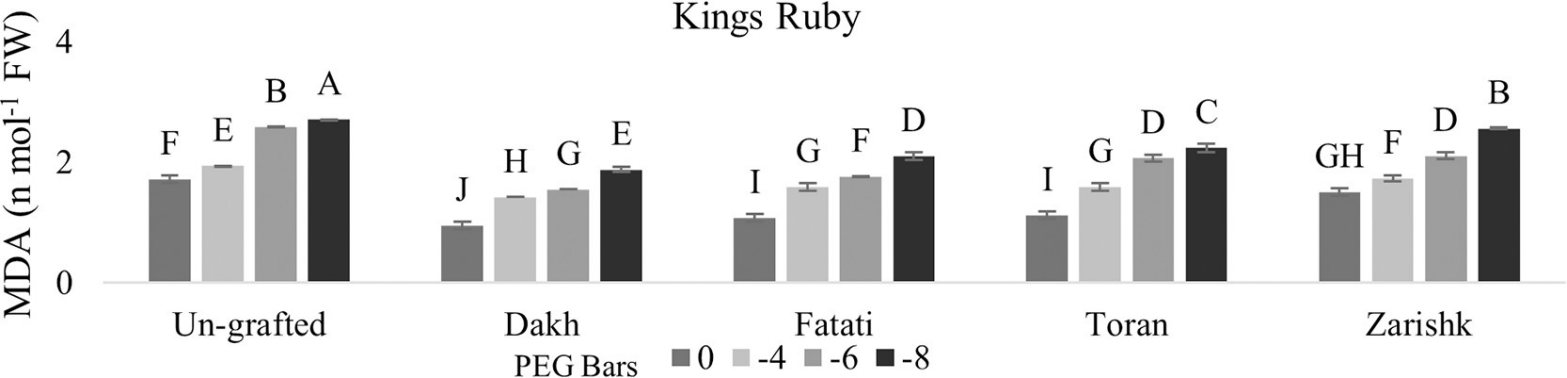
Malonaldehyde content (MDA) of un-grafted and grafted Kings Ruby scion variety on different wild grapevines under drought stress condition. Data is represented as mean SD and letters showed significant difference at p<0.05.

**Fig 4:**
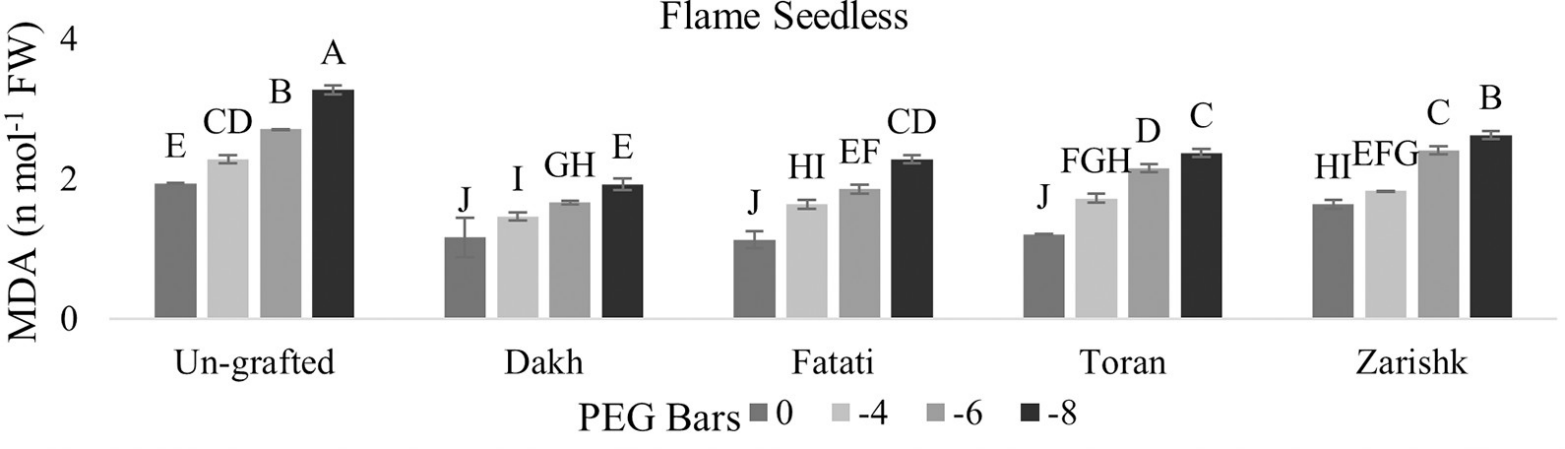
Malonaldehyde content (MDA) of un-grafted and grafted Flame Seedless scion variety on different wild grapevines under drought stress condition. Data is represented as mean SD and letters showed significant difference at p<0.05.

Statistical analysis related to MDA contents showed significant interaction between grafted plant and different level of drought stress at probability level less than 0.05 percent (Figs 3 and 4). Un-grafted commercial scion varieties showed higher rate of MDA contents under drought stress conditions due to the non-effective detoxification of ROS generation. Commercial scion varieties Kings Ruby and Flame Seedless showed significantly lower amount of MDA contents under drought stress conditions when grafted on different wild grapevines Dakh, Fatati and Toran as compared to un-grafted scion varieties, where MDA contents was higher due to more rapid activities of ROS. Malondialdehyde contents of scion variety Kings Ruby was much lower i.e., 2.49 *nmol*^−1^ when grafted on wild grapevine Zarishk.

### Stimulation of proline and glycine betaine accumulation in un-grafted and grafted plants

**Fig 5:**
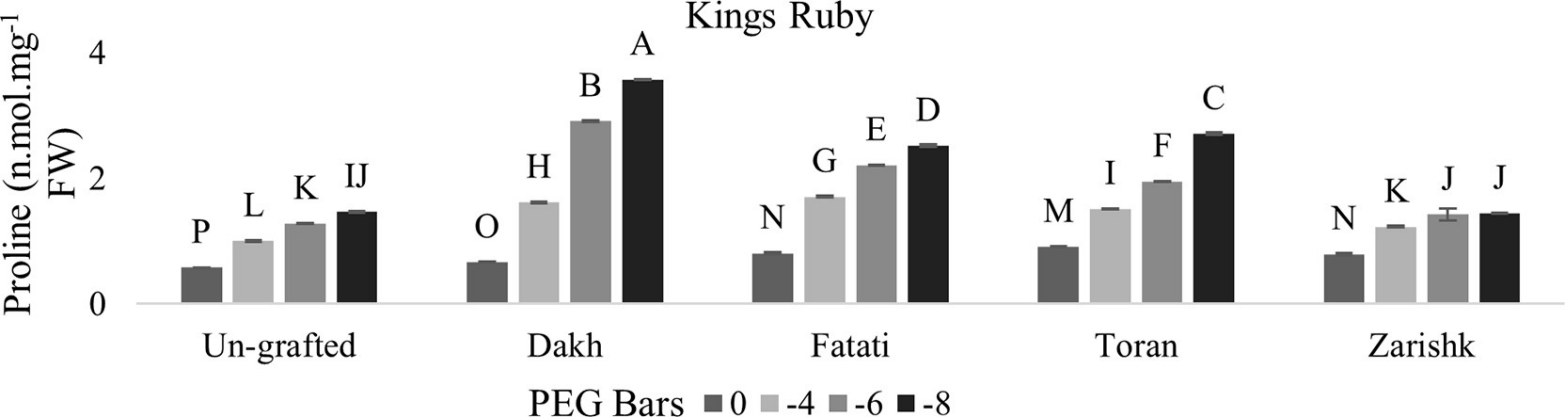
Proline content of un-grafted and grafted Kings Ruby scion variety on different wild grapevines under drought stress condition. Data is represented as mean SD and letters showed significant difference at p<0.05.

**Fig 6:**
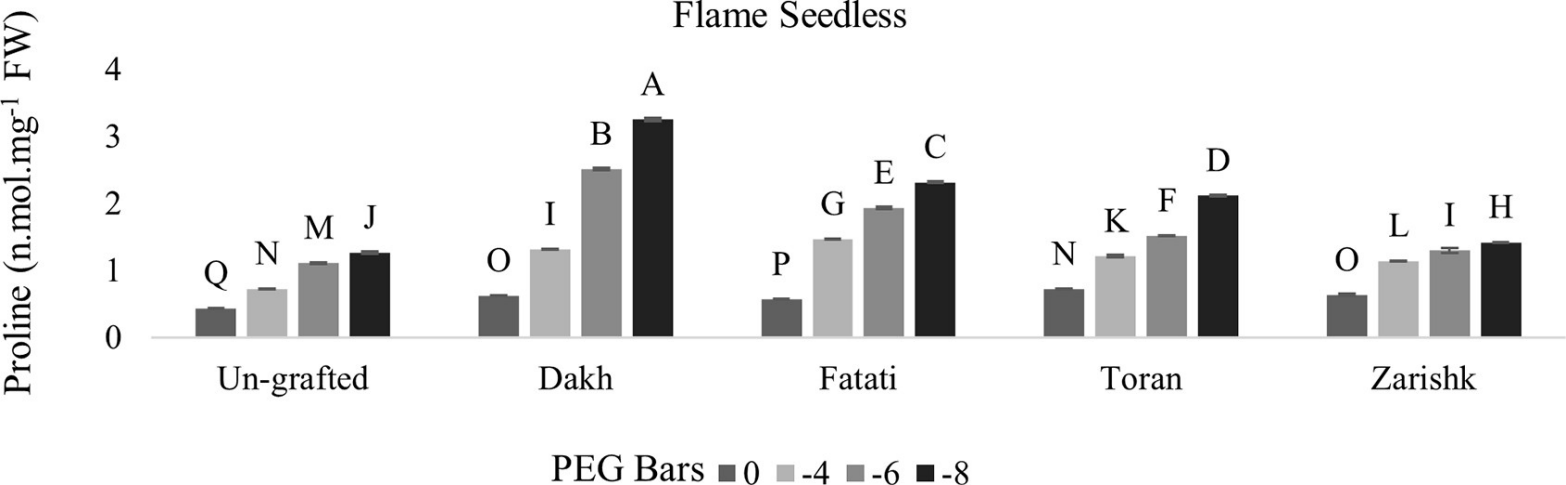
Proline content of un-grafted and grafted Flame Seedless scion variety on different wild grapevines under drought stress condition. Data is represented as mean SD and letters showed significant difference at p<0.05.

**Fig 7:**
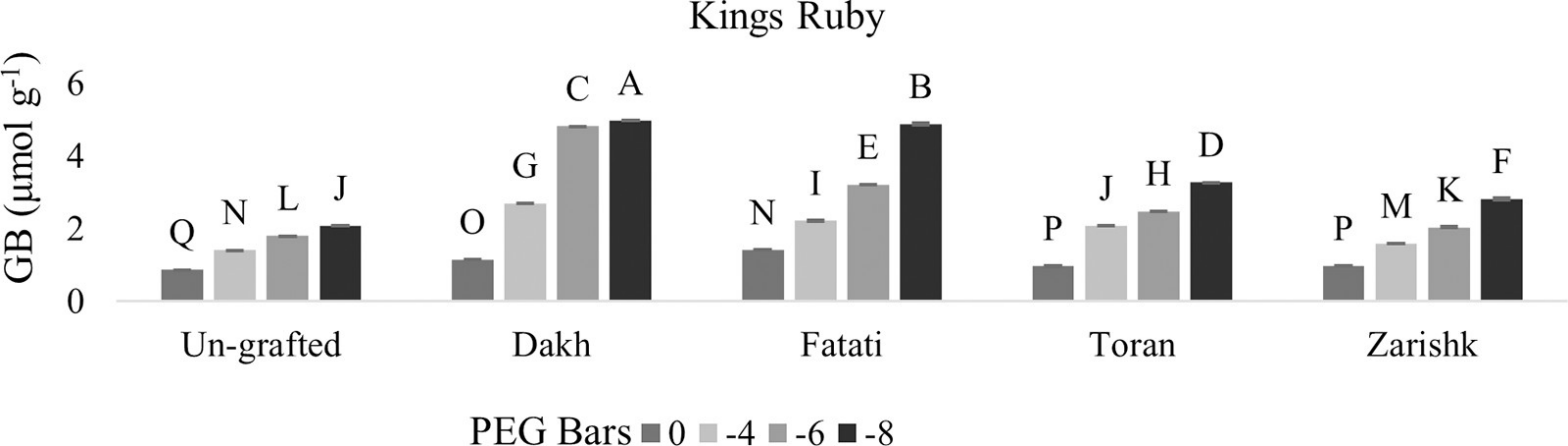
Glycine betaine content (GB) of un-grafted and grafted Kings Ruby scion variety on different wild grapevines under drought stress condition. Data is represented as mean SD and letters showed significant difference at p<0.05.

**Fig 8:**
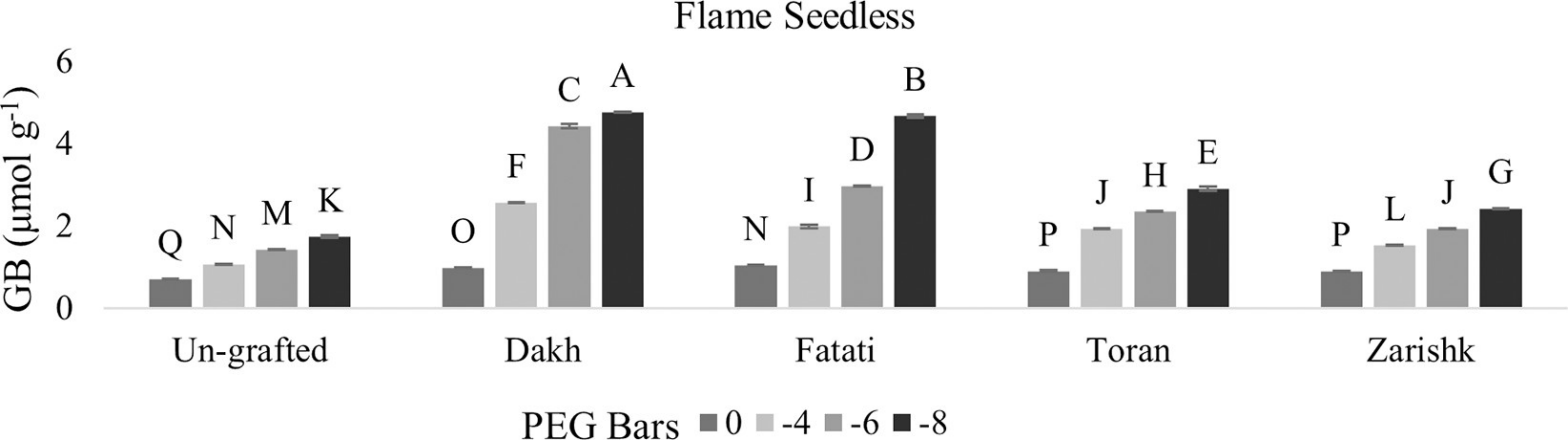
Glycine betaine content (GB) of un-grafted and grafted Flame Seedless scion variety on different wild grapevines under drought stress condition. Data is represented as mean SD and letters showed significant difference at p<0.05.

In most grafted wild grapevines proline and GB contents were increased significantly with increasing rate of drought stress whereas, among both osmolytes, GB increment was higher as compared to proline contents (Figs 5–8). When subjecting to different levels of drought stress, wild grapevines initiated the production and accumulation of proline and GB contents which increased constantly and reached to their peak level at -8 bars of drought stress. Due to the identified different tolerance levels of wild grapevines in previous study of Muawiya [21], commercial scion varieties were grafted on these wild grapevines to improve the osmotic adjustment of scion to counteract the negative effect of drought stress. Commercial scion variety Kings Ruby and Flame seedless grafted on wild grapevines Dakh and Fatati showed remarkable higher contents of proline and GB with progressive levels of drought stress. While wild grapevine Toran showed proline contents of 2.11 n.mol.mg^-1^ FW in Flame Seedless variety and 2.70 n.mol.mg^-1^ FW in Kings Ruby variety at -8 bars of drought stress. When comparing grafted plants with un-grafted commercial scion varieties Kings Ruby and Flame Seedless, it was clearly revealed that concentrations of proline and GB contents were increased significantly even in grafted scion varieties on relatively susceptible wild grapevine Zarishk.

### Enzymatic activities of un-grafted and grafted plants

#### Regulation of SOD and CAT activities by wild grapevines under drought stress

**Fig 9:**
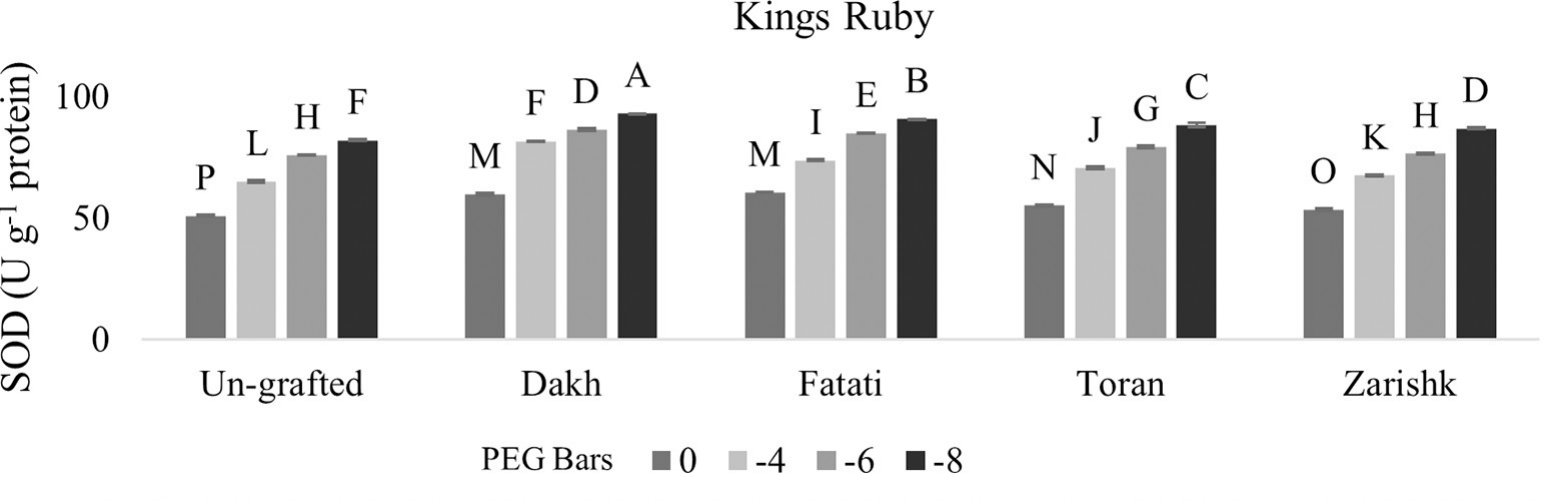
Superoxidase dismutase (SOD) enzymatic activity of un-grafted and grafted Kings Ruby scion variety grafted on different wild grapevines under drought stress condition. Data is represented as mean SD and letters showed significant difference at p<0.05.

**Fig 10:**
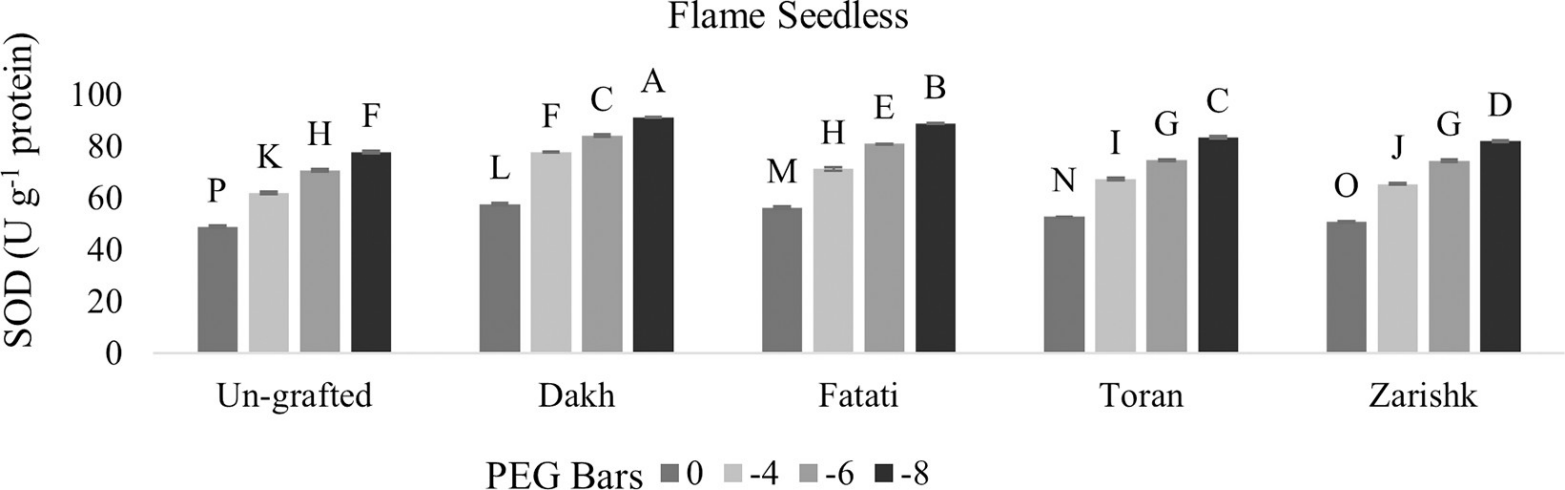
Superoxidase dismutase (SOD) enzymatic activity of un-grafted and grafted Flame Seedless scion variety grafted on different wild grapevines under drought stress condition. Data is represented as mean SD and letters showed significant difference at p<0.05.

**Fig 11:**
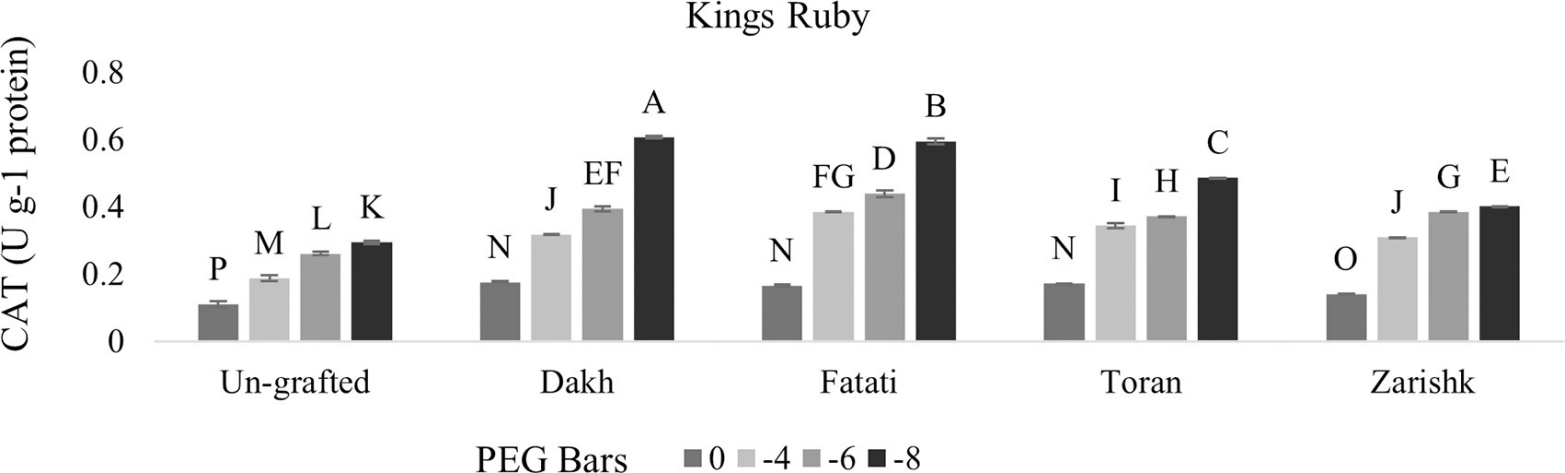
Catalase (CAT) enzymatic activity of un-grafted and grafted Kings Ruby scion variety grafted on different wild grapevines under drought stress condition. Data is represented as mean SD and letters showed significant difference at p<0.05.

**Fig 12:**
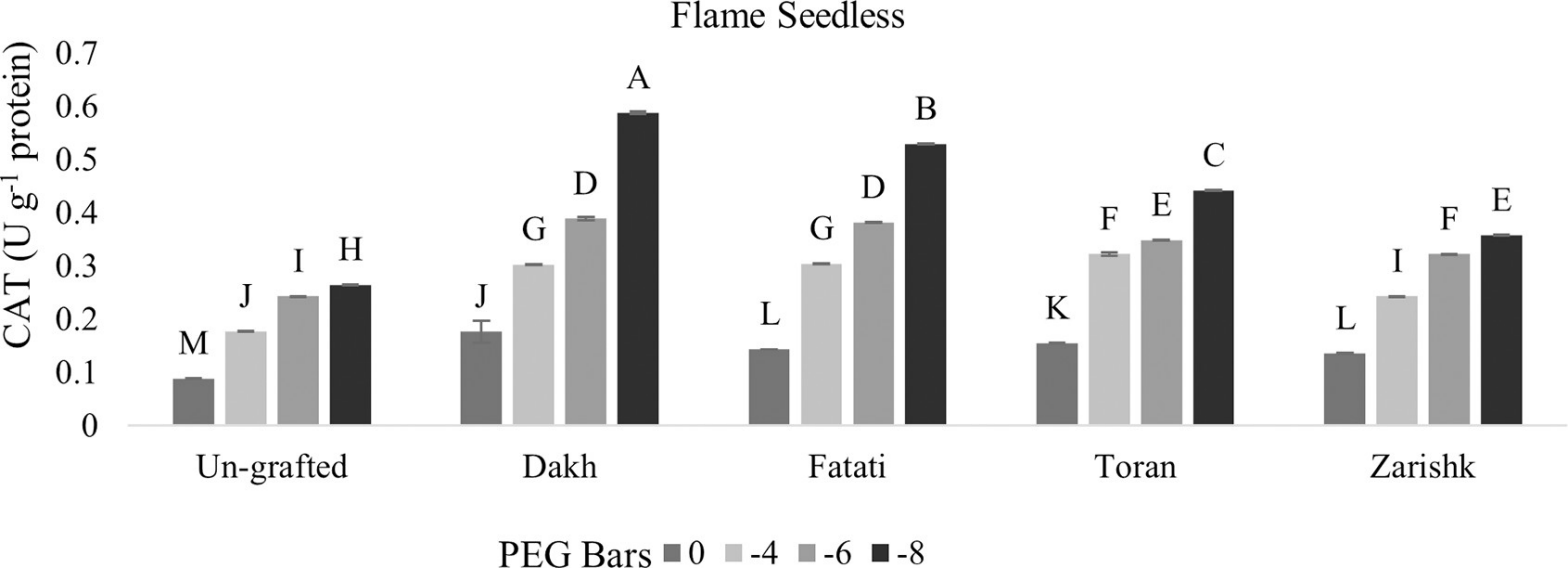
Catalase (CAT) enzymatic activity of un-grafted and grafted Flame Seedless scion variety grafted on different wild grapevines under drought stress condition. Data is represented as mean SD and letters showed significant difference at p<0.05.

Enzymatic activities were enhanced significantly in all grafted wild grapevines when drought stress level increased gradually but SOD activities were increased at higher rate as compared to CAT activities (Figs 9–12). Un-grafted plants of both commercial scion varieties showed susceptible behavior under drought stress condition, but Kings Ruby exhibited relatively improved rate of SOD and CAT activities especially at higher level of drought stress (-8 bars) as compared to Flame Seedless variety. Utilization of wild grapevines as rootstocks exhibited varying levels of tolerance in grafted commercial scion varieties therefore, at -8 bars of drought stress, higher level of SOD activity was observed in Kings Ruby variety grafted on relatively tolerant wild grapevines Dakh and Fatati. Superoxidase dismutase and catalase activities of grafted commercial scion varieties Kings Ruby and Flame Seedless were expressed to be improved by wild grapevine Toran as PEG induced drought stress increased from -4 bars to -8 bars. Relatively susceptible wild grapevine Zarishk showed least amount of antioxidant activities i.e., SOD and CAT enzymes in grafted commercial scion variety Flame Seedless when subjected to different levels of drought stress.

### Modulation of APX and POD activities by drought responsive wild grapevines

**Fig 13:**
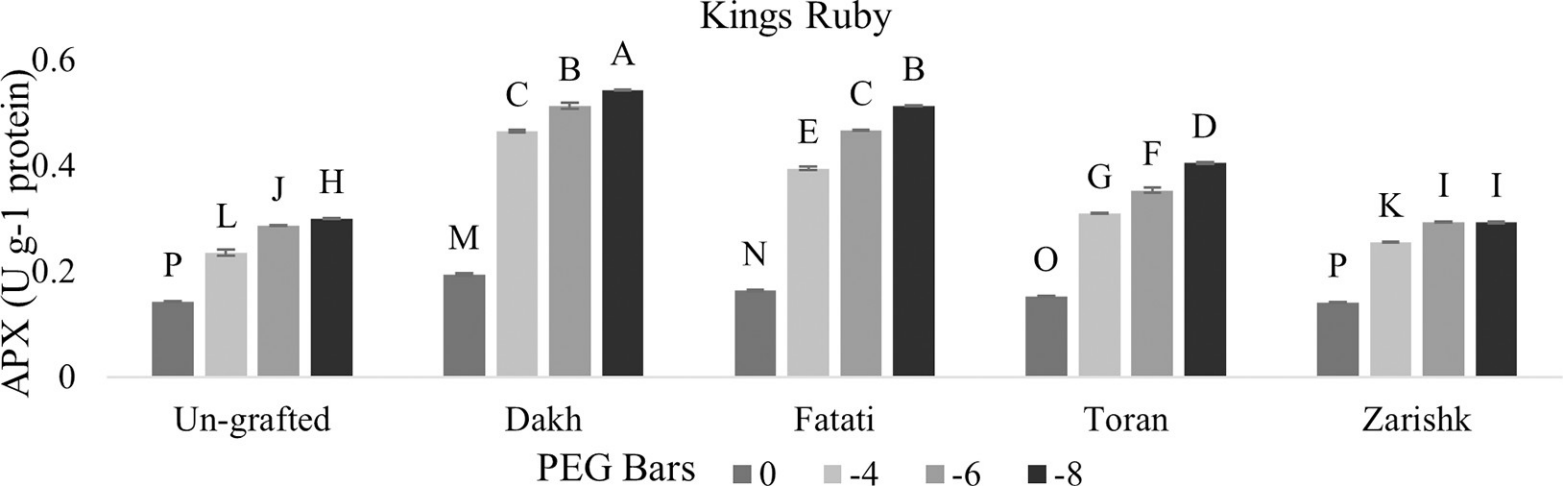
Ascorbate peroxidase (APX) enzymatic activity of un-grafted and grafted Kings Ruby scion variety grafted on different wild grapevines under drought stress condition. Data is represented as mean SD and letters showed significant difference at p<0.05.

**Fig 14:**
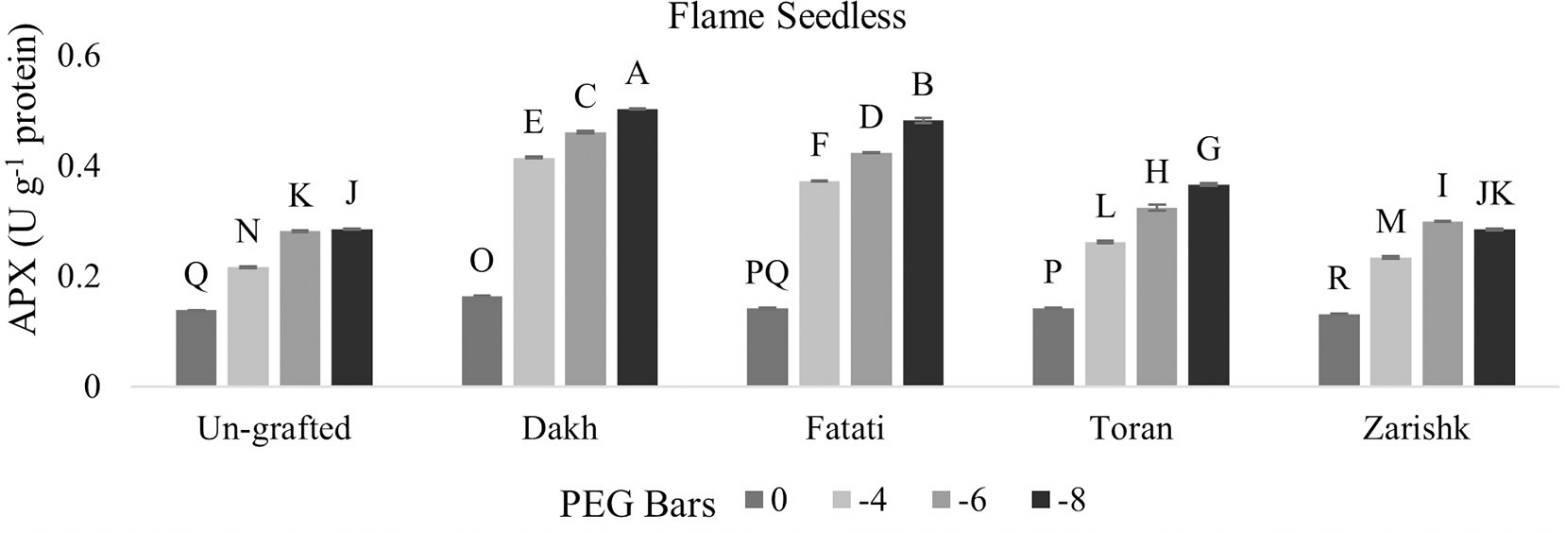
Ascorbate peroxidase (APX) enzymatic activity of un-grafted and grafted Flame Seedless scion variety grafted on different wild grapevines under drought stress condition. Data is represented as mean SD and letters showed significant difference at p<0.05.

**Fig 15:**
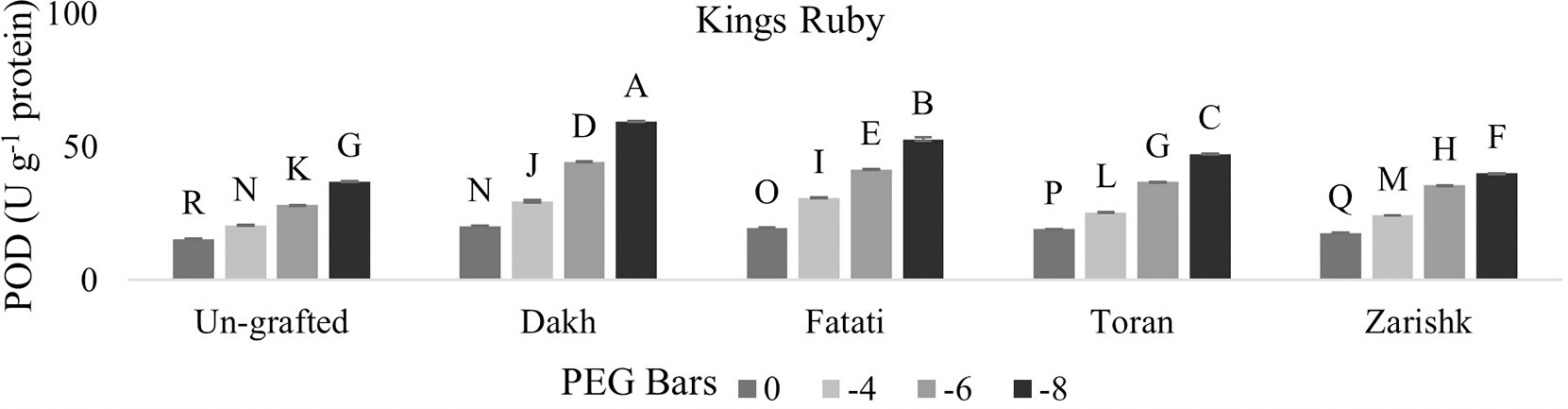
Ascorbate peroxidase (POD) enzymatic activity of un-grafted and grafted Kings Ruby scion variety grafted on different wild grapevines under drought stress condition. Data is represented as mean SD and letters showed significant difference at p<0.05.

**Fig 16:**
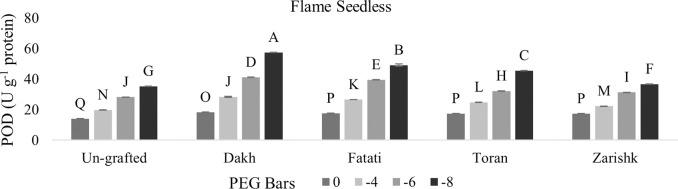
Ascorbate peroxidase (POD) enzymatic activity of un-grafted and grafted Flame Seedless scion variety grafted on different wild grapevines under drought stress condition. Data is represented as mean SD and letters showed significant difference at p<0.05.

Enzymatic activities of APX and POD were increased significantly in all grafted wild grapevines with increasing rate of drought stress (Figs 13–16). Grafting has been extensively used to advance the tolerance abilities of scion varieties against oxidative stress and ultimately to sustain the productivity of grafted plants under drought stress conditions. Commercial scion varieties Kings Ruby and Flame Seedless showed increased rate of enzymatic activities, particularly APX at -6 bars of drought stress, when grafted on relatively tolerant wild grapevines Dakh and Fatati. Moderate level of tolerance attributed by APX, and POD enzyme activities were induced by wild grapevine Toran in commercial scion variety Kings Ruby when subjected to -8 bars of drought stress. Least level of APX and POD enzymes were exhibited by commercial varieties when grafted on relatively susceptible wild grapevine Zarishk.

### Bi-plot analysis

**Fig 17:**
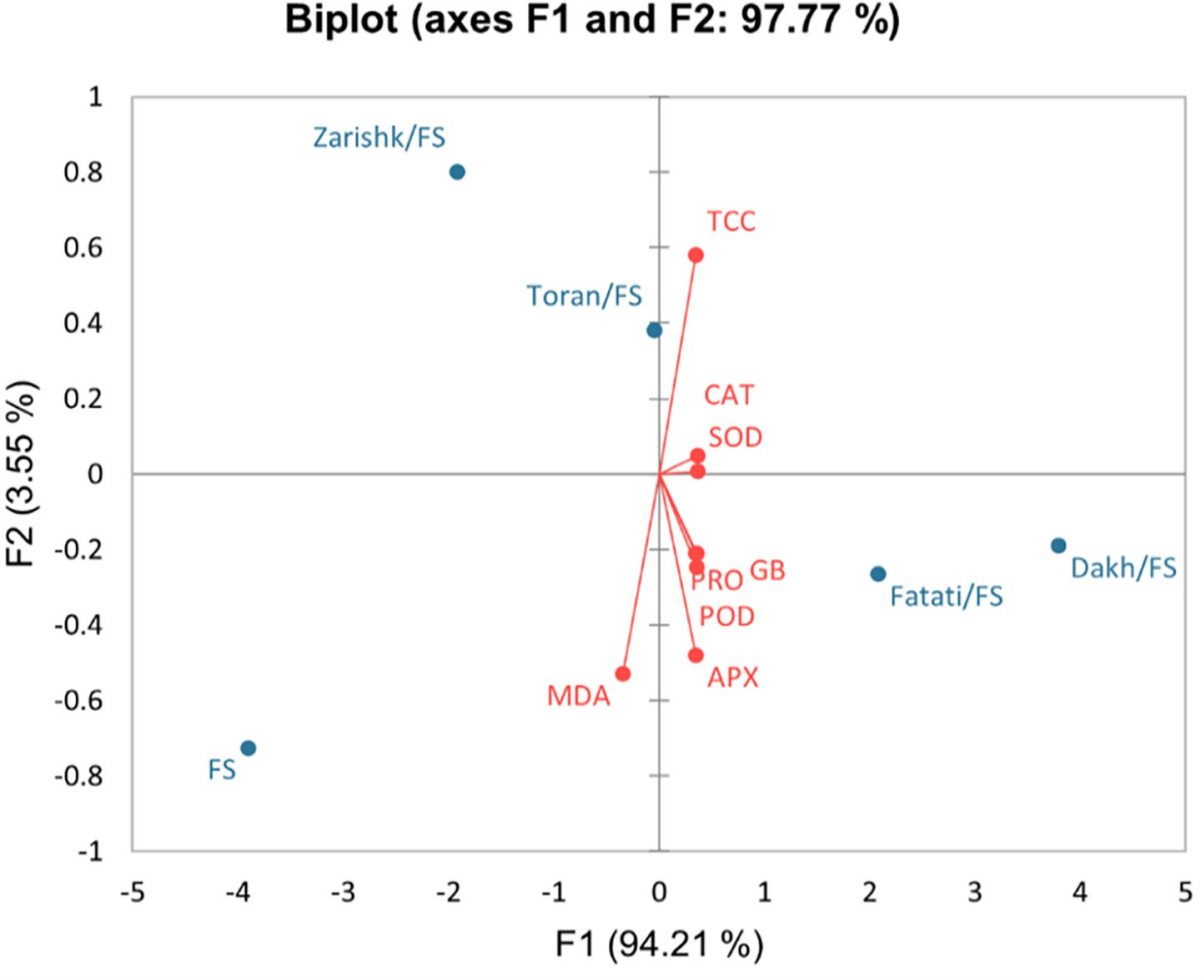
Bi-plot graph of the first (PC1) and second (PC2) principal components of enzymatic and non-enzymatic biochemical traits in un-grafted and grafted Flame Seedless scion variety on different wild grapevines under -8 bars of drought stress.

**Fig 18:**
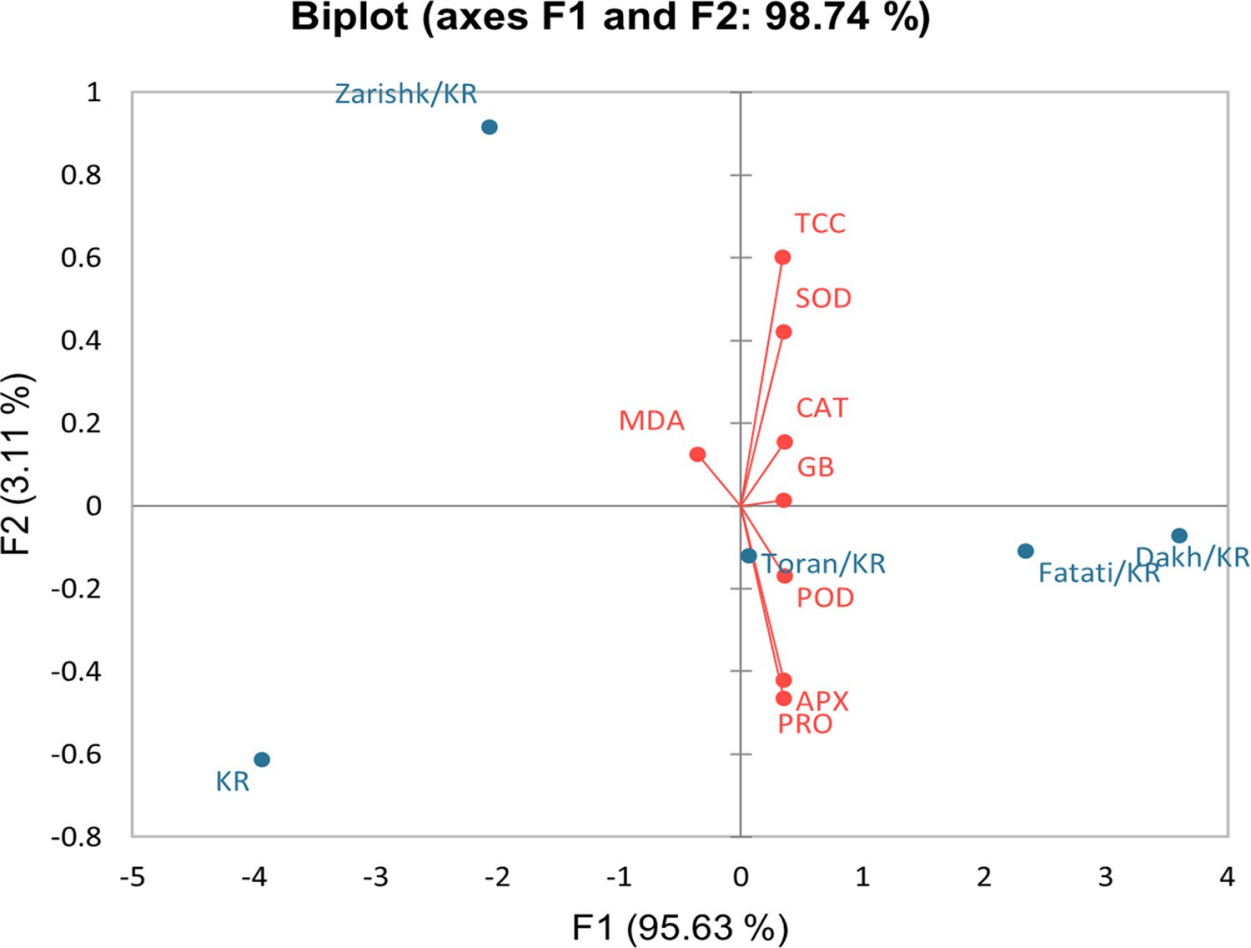
Bi-plot graph of the first (PC1) and second (PC2) principal components of enzymatic and non-enzymatic biochemical traits in un-grafted and grafted Kings Ruby scion variety on different wild grapevines under -8 bars of drought stress.

Bi-plot graphs, which were drawn based on the first and the second components, were used to identify the most and least tolerant wild grapevines as rootstock (Figs 17 and 18). The PC1 and PC2 specified the enzymatic and non-enzymatic bio-chemical traits in different groups among which PC1 of Flame Seedless (94.21%) and Kings Ruby (95.63%) showed the largest group of positive values with TCC, proline, GB, SOD, CAT, POD and APX while PC2 of Flame Seedless (3.55%) and Kings Ruby (3.11%) represent the negative values that includes oxidative stress parameter i.e., MDA content. Therefore, PC1 factor was known as enzymatic and non-enzymatic defense system and alone can be considered to identify the most and least drought tolerant wild grapevines as rootstock. According to the bi-plot graphs, wild grapevines Dakh and Fatati were identified as relatively drought tolerant rootstocks while wild grapevine Zarishk were considered as relatively drought susceptible rootstocks. Bi-plot graphs also showed that grafted wild grapevine Toran have somehow neutral trend along with bi-plot and observed as near to their origin in positive relation which makes them relatively moderate drought tolerant rootstock.

### Cluster analysis

**Fig 19:**
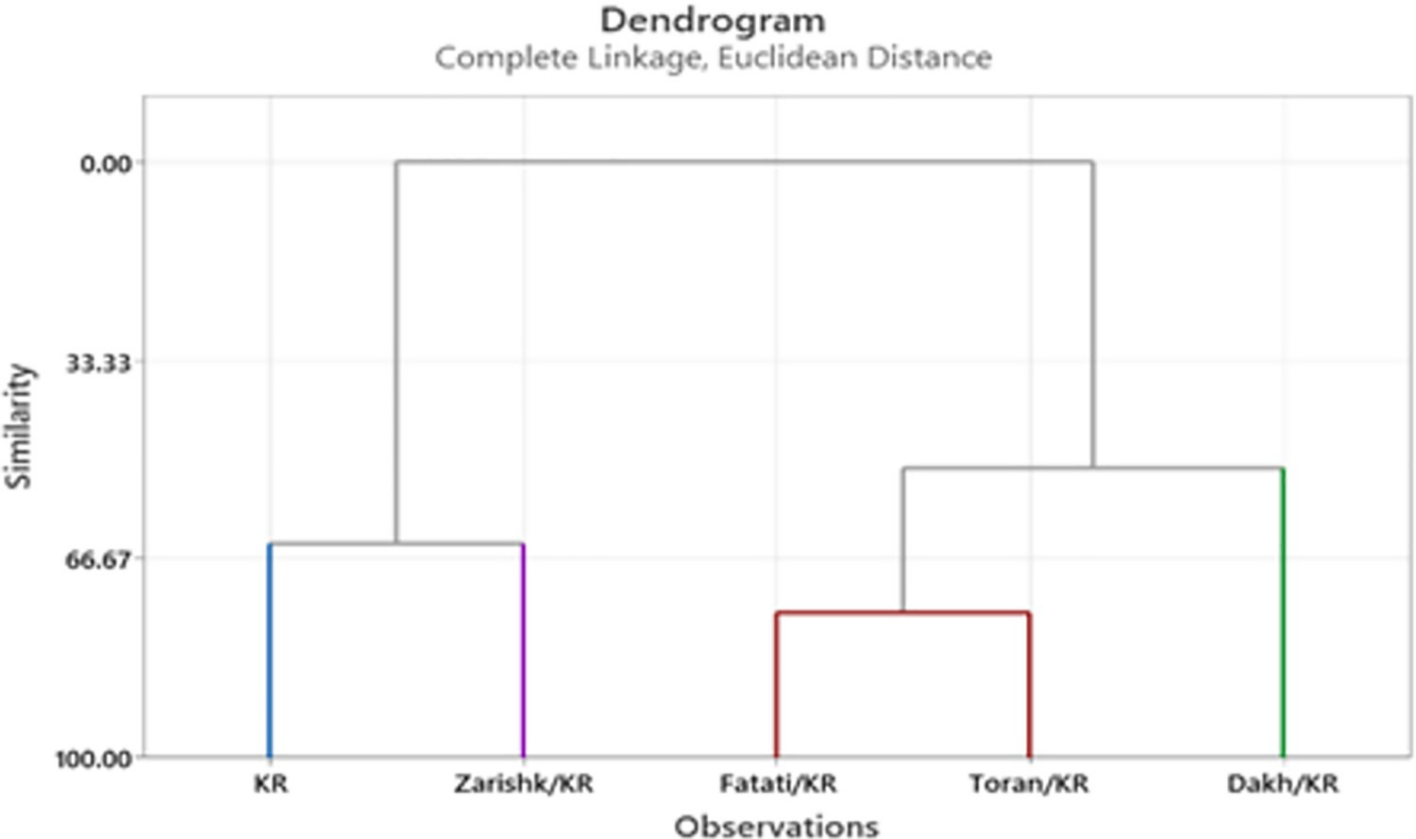
Dendrogram of cluster analysis of un-grafted and grafted Kings Ruby scion variety based on enzymatic and non-enzymatic biochemical traits under -8 bars of drought stress.

**Fig 20:**
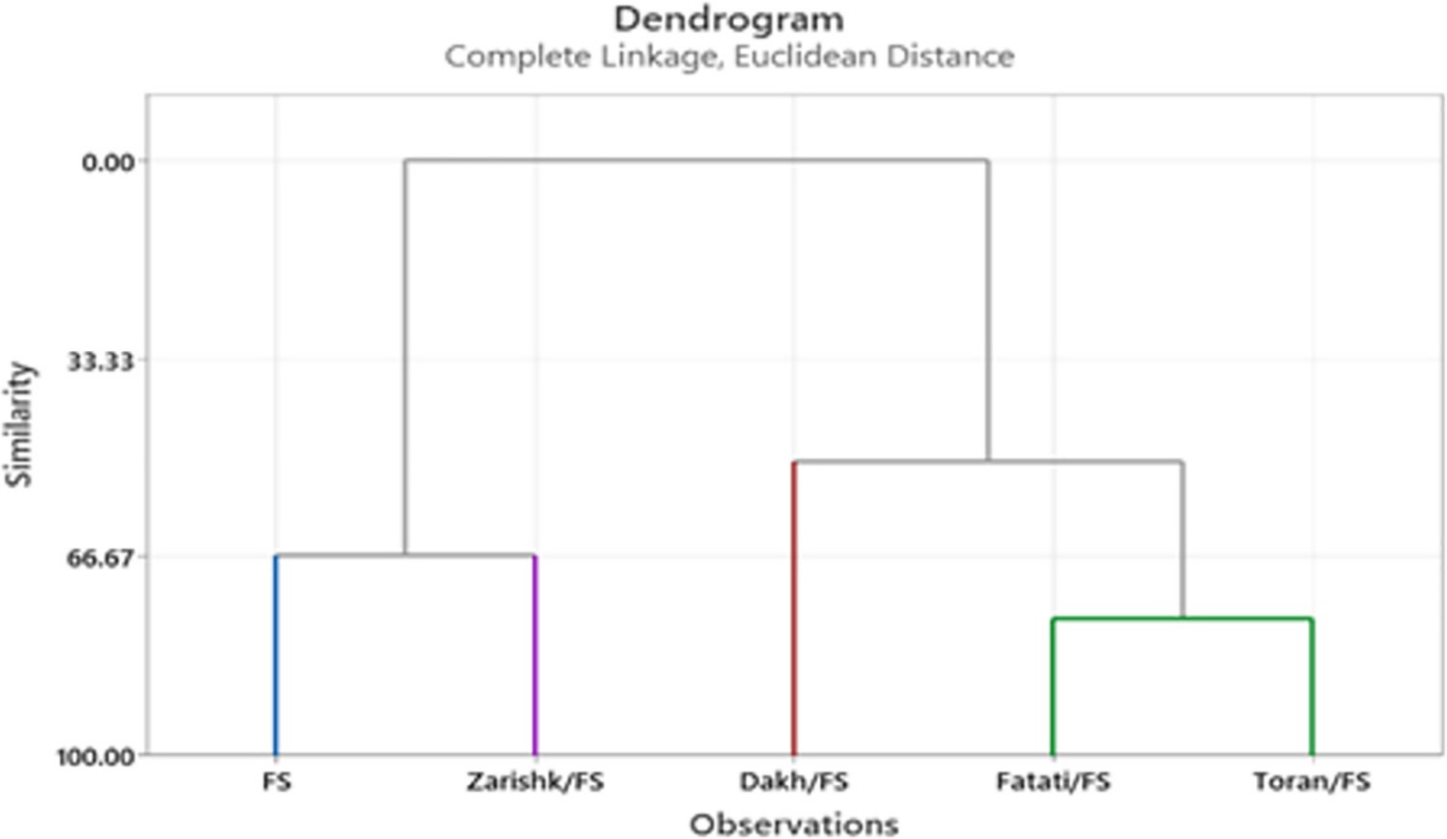
Dendrogram of cluster analysis of un-grafted and grafted Flame Seedless scion variety based on enzymatic and non-enzymatic biochemical traits under -8 bars of drought stress.

Cluster analysis was used to group the wild grapevines as rootstock under severe (-8 bars) level of drought stress conditions. According to the dendrograms at -8 bars of drought stress, wild grapevines Dakh showed more similarity with Fatati and Toran while a distant relation of these wild grapevines was observed with commercial scion varieties Flame Seedless and Kings Ruby (Figs 19 and 20). Wild grapevine Toran showed some similarity level with wild grapevines Dakh and Fatati. Closest association of wild grapevine Zarishk was observed with commercial scion variety Flame Seedless. Hence, from cluster analysis, we can categorize grafted wild grapevines Dakh and Fatati into relatively tolerant class, while commercial scion varieties Kings Ruby and Flame Seedless and wild grapevine Zarishk into relatively susceptible class.

### Visual observations of treated ungrafted and grafted commercial scion varieties on different wild grapevines

**Fig 21:**
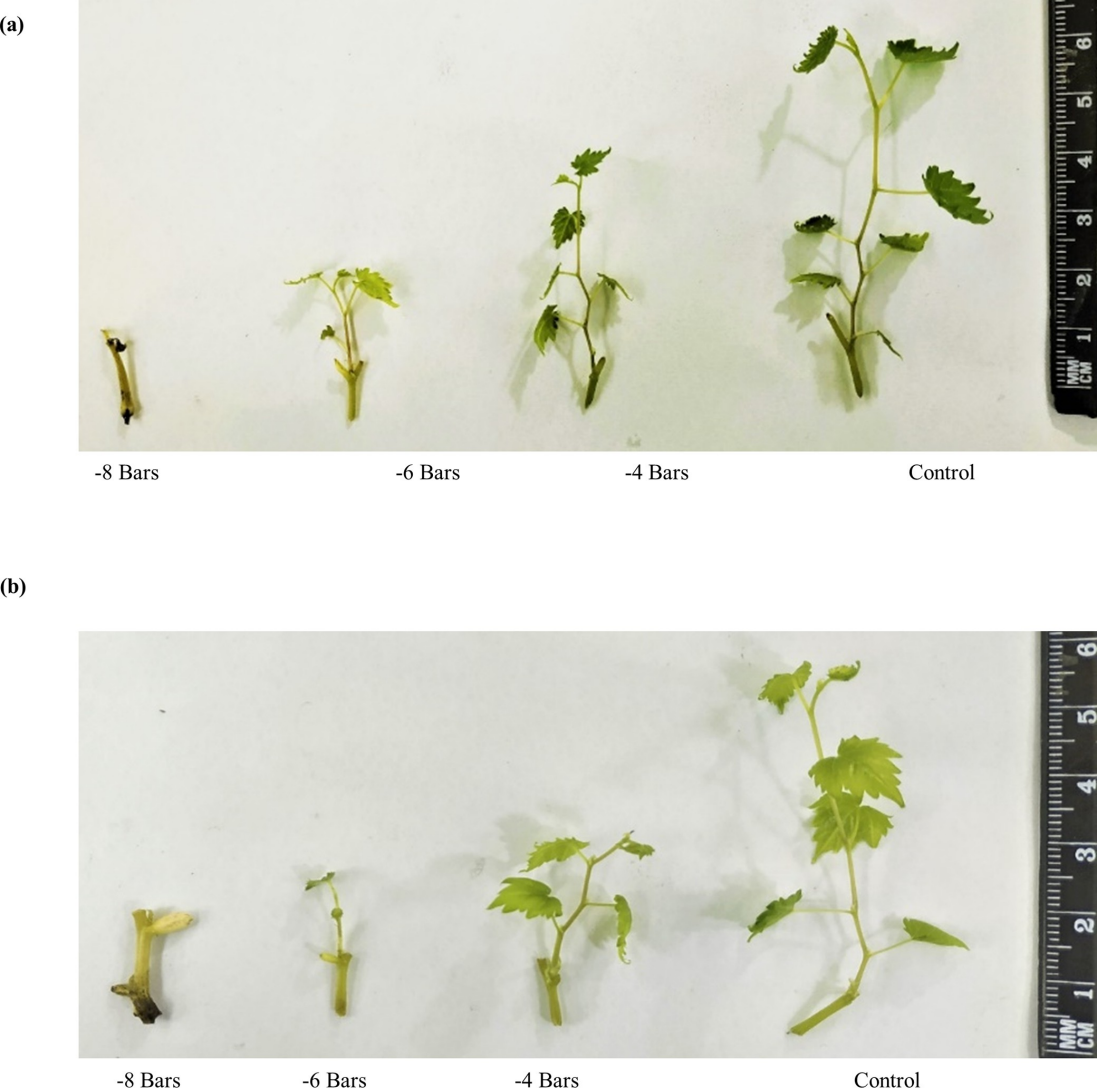
Treated un-grafted commercial scion varieties Kings Ruby (a) and Flame Seedless (b).

**Fig 22:**
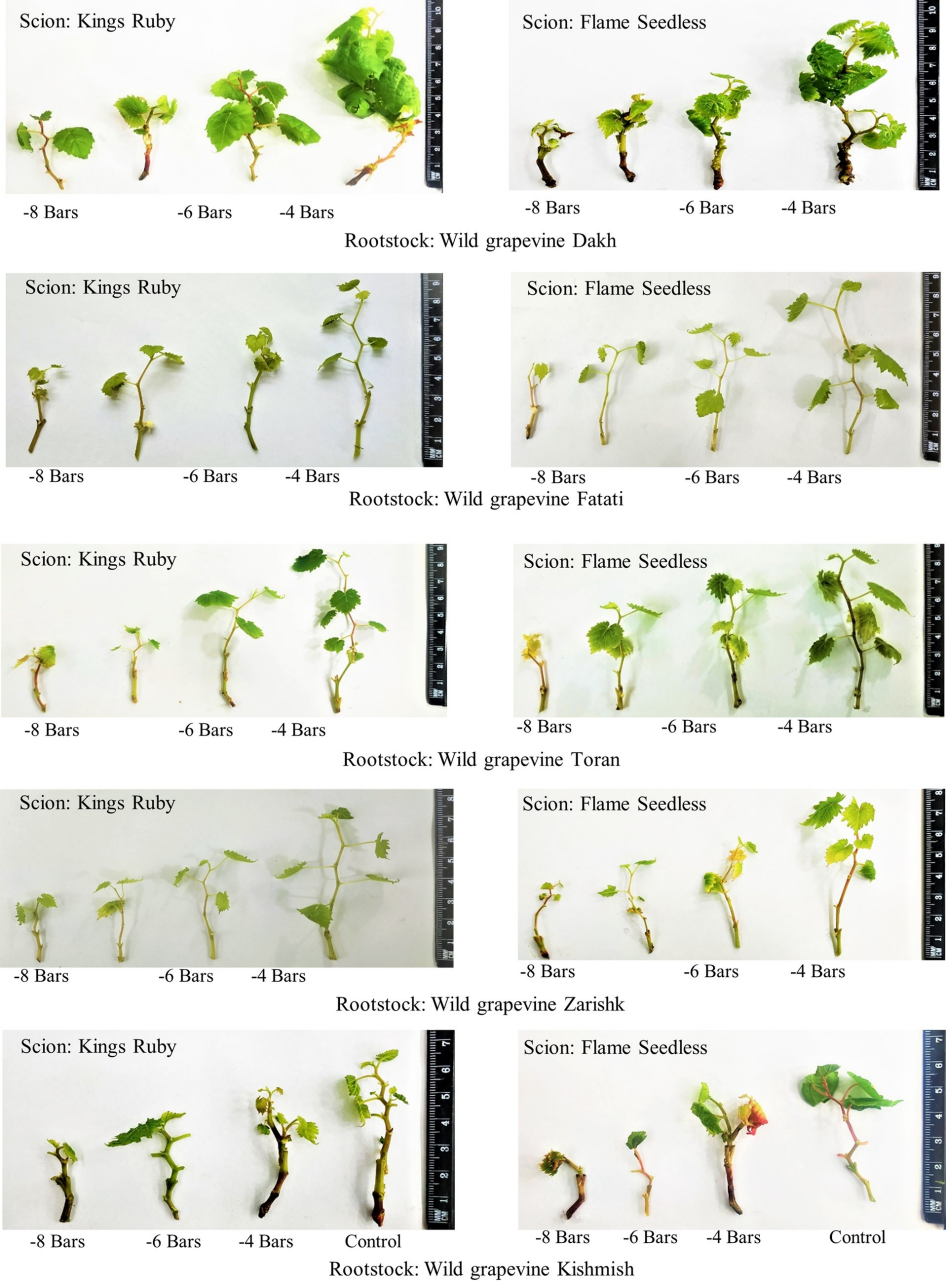
Treated plants of grafted commercial scion varieties with different wild grapevines.

Visual observations of ungrafted commercial scion varieties showed less growth and number of leaves due to the higher susceptibility towards drought stress and less regulation of enzymatic and non-enzymatic activities (Fig 21). When these commercial scion varieties were grafted on different wild grapevines, healthier growth with lush green leaves were observed due to the better defensive mechanism of biochemical activities which were upregulated by wild grapevines and results in reduced effect of drought stress on grafted scion varieties (Fig 22).

## Discussion

Drought stress damages the chlorophyll content in commercial scion varieties by causing photo-oxidation and degradation of pigment molecules which affect the efficiency of light harvesting ability of varieties. Degradation of chlorophyll content in grafted commercial varieties Kings Ruby and Flame Seedless could be associated with the higher production of reactive oxygen species and decreased rate of photosynthetic potential in the leaf cells. This hypothesis was confirmed by Liu et al. [29] in walnut Juglans rootstock, where reduction of chlorophyll content, either with less synthetization or rapid rate of breakdown, was observed due to the lower photosynthesis rate and production of oxidative stress which ultimately damage the green chlorophyll pigment. Relatively more loss of chlorophyll content was observed at higher level of drought stress in commercial scion varieties grafted on moderately tolerant wild grapevine Toran which might be linked with the upregulation of photosynthetic photoprotection mechanism that can be regulated by the genes related to the light reactions. This mechanism of photoprotection was also upregulated under drought stress condition by citrus Rangpur lime rootstock in grafted sweet orange through the down regulation of genes related to light reactions such as CAB, PSBO, PSBP etc. These genes help grafted sweet orange to reduce its chlorophyll content in its leaves which dropdown the ability of leaves toward light absorption and subsequently also reduce the risk of ROS [30]. Wild grapevine Zarishk showed relatively susceptible behavior to drought stress but when grafted with commercial scion varieties, it yields significant results even at higher stress level as compared to un-grafted plants. Among both grafted commercial varieties, Kings Ruby scion variety performed better when grafted on wild grapevine Zarishk as compared to Flame Seedless variety where chlorophyll content was reduced drastically with increasing rate of drought stress. Severe loss of chlorophyll content in commercial variety Flame Seedless grafted on wild grapevine Zarishk might be due to the lower rate of fluorescence emitted from the chlorophyll which represent the less photosynthetic energy conversion in leaf cells. Reduction of chlorophyll content in grafted citrus sweet orange was also expressed by the grafted citrus ‘Sunki Tropical’ rootstock where disbalance between lower fluorescence and variable florescence was observed due to the reduced content of green pigment in the scion variety [31]. Similarly higher chlorophyll content of grafted grapevine K7 clone of Sultani Seedless cultivar was observed by Sucu et al. [14] when grafted on 110R, 41B, and 420A rootstocks.

The degree of damage caused by oxidative stress most often can be observed by monitoring changes in production of MDA concentrations inside the plant cells and lower concentrations of MDA contents in cells can be associated with the better tolerance ability of plants against drought stress. Increased ROS generation and MDA contents have negative effect on metabolic activities which plays key role to boost up the immune system of plant to resist the different stress conditions. Wild grapevines Dakh and Fatati reduced the MDA contents in commercial scion varieties which might be due to the effective metabolic activities and sustainability of relative water contents in grafted varieties at higher level of drought stress. Our findings are in line with the observations of Hussain et al. [32] as they also observed that increased relative water content by drought tolerant citrus Carrizo citrange rootstock lower the MDA accumulation due to the enhance antioxidant activities assisted by the effective metabolic activities which improve its tolerant ability. Antioxidant activities have major role to decrease the MDA contents which might be activated by relatively tolerant wild grapevines Dakh and Fatati and subsequently induced tolerance in grafted commercial scion varieties due to the scavenging of ROS produced by drought stress. Higher rate of antioxidant activities was also observed by Goharrizi et al. [33] in drought tolerant pistachio rootstocks UCB-1 and Badami which have leading role in scavenging the superoxide ion and subsequently reducing the MDA contents. Several WRKY related transcriptional factors might be another responsible factor for the reduction of MDA contents in commercial scion varieties Kings Ruby and Flame Seedless which have also been observed by Haider et al. [34] to be upregulated in drought tolerant wild peach (*Amygdalus persica*) Batsch rootstock.

Several organic compounds such as sugars, proline, and glycine betaine (GB) act as prime osmotica which helps plants in osmotic adjustment during drought stress conditions [35]. Osmolytic compounds i.e., proline and glycine betaine (GB) contents have strong positive relation with enzymatic activities and to combat the increased level of drought stress, relatively drought tolerant wild grapevines Dakh and Fatati upregulate some drought related enzyme activities which may enhanced the protection of osmolyte synthetization from suppression and the chlorophyll degradation in grafted commercial scion varieties Kings Ruby and Flame Seedless. Our findings of strong relation were corroborated with enhanced activities of drought responsive enzymes such as glutamate kinase and glutamate ligase that were also showed to be upregulated by drought tolerant citrus Mexican lime rootstock which plays key role in improving the production of osmolytes in grafted citrus Kinnow Mandarin scion variety by maintaining the chlorophyll under drought stress conditions [36]. Moreover, wild originated grapevine 110R rootstock significantly increased the accumulation of organic solutes grapevine grafted Superior Seedless cultivar. However, grapevine SO4 rootstock was showed susceptible response to grafted variety under drought stress conditions [37].

Plants also develop several antioxidative system which reduce the negative effect of ROS accumulation by detoxify them into less toxic compounds [38, 39]. To eliminate and degrade harmful oxygen species at early stage, SOD and CAT are considered as most active enzymes that maintain oxidative equilibrium during stress condition [40]. Catalase activity had positive relation with SOD enzyme, therefore, wild grapevines upregulated the antioxidative enzymes such as SOD and CAT at earlier stage of stress to scavenge the production of oxygen free radicals and subsequently degradation of dismutase product e.g., H_2_O_2_. Increased level of SOD and CAT activities were observed in commercial scion varieties Kings Ruby and Flame Seedless when grafted on relatively tolerant wild grapevines Dakh and Fatati possibly due to the upregulation of certain genes by wild grapevines which enhance the activities of SOD and CAT enzymes in grafted scion varieties. Expression of ‘SOD; Cu-Zn’ and ‘CAT1’ genes encoded with ROS scavenging system were also observed by Haider et al. [34], which were upregulated by the drought tolerant wild peach (*Amygdalus persica* L.) Batsch rootstock that subsequently increased the activities of SOD and CAT enzymes in the leaves of peach Yoshihime variety. Under drought stress conditions several other differentially expressed genes related to SOD and CAT activities i.e., ‘redox. dismutases and catalases genes were also showed to be upregulated by another drought tolerant citrus Rangpur rootstock to efficiently control the oxidative stress and cellular hemostasis of grafted drought susceptible sweet orange variety [31]. Under higher level of drought stress several protein classes mainly the NAC family was might also be the responsible reason for improved rate of SOD and CAT activities in commercial scion varieties grafted on relatively tolerant wild grapevines Dakh and Fatati. Jia et al. [41] also analyzed the expression patterns of different proteins encoded with SOD and CAT enzymes and revealed that MdCAT, and MdSOD proteins were expressed by drought tolerant apple rootstock i.e., *Malus hupehensis*. var. Pingyiensis which ultimately increased the rate of antioxidant enzymes to scavenge the ROS production. Ascorbate peroxidase enzyme utilizes the Ascorbate-Glutathione (AsA) cycle to convert the toxic H_2_O_2_ into water molecules, whereas POD utilizes the several reductants that are readily available in the cells i.e., sugar or sucrose etc. [34]. Wild originated drought tolerant grapevine rootstocks 140Ru and 110R also exhibited higher rate of enzymatic activities in both control and drought stress condition as compared to drought sensitive 5BB and SO4 rootstocks [14].

Excessive generation of ROS can be dismutase by CAT enzyme but due to the strong relation of CAT with APX and POD activities, wild grapevines upregulate the APX and POD enzymes to reduce the toxic effect of H_2_O_2_ [42]. Commercial scion varieties Kings Ruby and Flame Seedless showed increased rate of enzymatic activities particularly APX when grafted on relatively tolerant wild grapevines Dakh, Fatati and Toran. This increased level of enzymatic activities might be associated with the strong transcription responses that could be upregulated effectively by relatively tolerant wild grapevines through the perception of signals and electrons transportation that might be generated from the imbalance redox state. The improved upregulation of transcriptional responses related to the enzymatic activities such as cAPX was also observed by Dos Santos et al. [43] in drought tolerant grafted citrus Rangpur Santa plants as compared to drought susceptible citrus Sunki Maravilha plants when subjected to moderate level of drought stress. Expression of cAPX transcript was controlled by the redox state of electrons and H_2_O_2_ generation inside the cell compartments. Genetic intonations of different wild grapevines to upregulate the expressions of different genes were also corroborated by Balfagon et al. [44] in citrus Carrizo rootstock. Ascorbate peroxidase activity of citrus rootstock was triggered by the expression of APX2 gene which dismutase the H_2_O_2_ by using the ascorbate-glutathione cycle as electron donor. Similar findings were also exhibited by Zandalinas et al. [45] in the same citrus drought tolerant Carrizo rootstock due to the expression of another APX1 gene which upregulate the activities of cytosolic APX that subsequently helps the grafted plants to acclimatized under drought stress condition. Healthy growth and survival of grafted scion varieties are generally linked with the less drought damaging effect which might be associated with the increased rate of enzymatic activity through the relatively tolerant wild grapevines that can efficiently upregulate the cellular activities and subsequently reduce the production of ROS in the shoot and leaves of scion. Increased enzymatic activities were also observed by Santos et al. [39] in drought tolerant citrus Rangpur lime and Citrus sunki rootstocks, which were attributed by the efficiently upregulation of APX enzyme activities that assist the elastic properties of grafted ‘Citrus latifolia Tanaka’ cell wall which increased the plant shoot growth under drought stress conditions. Similarly enzymatic activities of peroxidase were also reported to be upregulated significantly in Flame Seedless cultivar when grafted on relatively drought tolerant grapevine rootstock Paulsen [46].

## Conclusions

Enzymatic and non-enzymatic biochemical responses of wild grapevines were found as key indices against different levels of PEG-6000 induced drought stress and based on these markers, wild grapevines Dakh (*Vitis vinifera* subsp. *sylvestris*) and Fatati (*Vitis vinifra* subsp. *sativa*) were identified as relatively tolerant rootstocks, Toran (*Vitis vinifera* subsp. *sylvestris*) as relatively moderate tolerant rootstock whereas Zarishk (*Vitis vinifera* subsp. *sylvestris*) as relatively susceptible rootstock. These identified different tolerance levels of wild grapevines as rootstock can be further utilized in viticulture industry for breeding program to develop a sustainable viticulture under ongoing climate changes and global water scarce condition.
